# Infectious diseases and predominant travel-related syndromes among long-term expatriates living in low-and middle- income countries: a scoping review

**DOI:** 10.1186/s40794-022-00168-4

**Published:** 2022-05-01

**Authors:** Amornphat Kitro, Thundon Ngamprasertchai, Kriengkrai Srithanaviboonchai

**Affiliations:** 1 Department of Community Medicine, Faculty of Medicine, Chiang Mai University, Chiang Mai, 50200 Thailand; 2 Department of Clinical Tropical Medicine, Faculty of Tropical Medicine, Mahidol University, Bangkok, 10400 Thailand; 3 Research Institute for Health Sciences, Chiang Mai University, Chiang Mai, 50200 Thailand

**Keywords:** Infectious related health problems; Expatriate; Long-term Traveler; Travel Medicine Practitioner, low-and middle-income country

## Abstract

**Introduction:**

Expatriates working in low-and middle-income countries have unique health problems. Migration leads not only to an increase in individual health risk but also a risk of global impact, such as pandemics. Expatriates with no prior experience living in tropical settings have expressed greatest concern about infectious diseases and appropriate peri-travel consultation is essential to expatriates. The objective of this review is to describe infections and travel-related syndromes among expatriates living in low-and middle-income countries.

**Methods:**

MEDLINE database since the year 2000 was searched for relevant literature. Search terms were “long-term travel”, “expatriate”, and “health problems”. The additional references were obtained from hand-searching of selected articles.

**Results:**

Up to 80% of expatriates suffered from gastrointestinal problems followed by dermatologic problems (up to 40%), and febrile systemic infection/vector-borne/parasitic infection (up to 34%) Expatriates living in Southeast Asia were at risk of vector-borne diseases including dengue and non-*Plasmodium falciparum* (*pf*) malaria while expatriates living in South Asia had a high prevalence of acute and chronic diarrhea. Staying long-term in Africa was related to an elevated risk for *pf* malaria and gastrointestinal infection. In Latin America, dermatologic problems were commonly reported illnesses among expatriates.

**Conclusion:**

Certain health risks for expatriates who are going to depart to specific regions should be the focus of pre-travel consultation. Specific health preparations may reduce the risk of disease throughout their time abroad. Disease and symptom awareness is essential for screening, early diagnosis, and better health outcomes for ill-expatriates.

## Introduction

A long-term traveler typically is defined as any traveler visiting a foreign country for longer than six months for any reason [1]. Some experts classified long-term travelers by their travel reasons such as business, diplomat, volunteer, field-based research, corporate employees, military personnel, digital nomad, missionaries, and retirees [2]. Expatriates are a subgroup of those long-term stay travelers living outside their home country mostly for occupational purposes with definite plans [1, 3, 4]. During their stay, expatriates have to use local infrastructures and are exposed to the hazards of the destination country. Traveling by expatriates has become a contributory factor to the global spreading of infectious diseases [1, 3, 4]. The chance of contracting illness increases with the longer the time spent abroad. People who plan to travel to stay in other countries for a long period of time need proper health preparation to reduce possible risk of illnesses during their stays overseas [5–8].

Types and extent of health issues among expatriates are influenced by travel destination, duration of stay, and travel itinerary [1]. Infectious diseases, non-communicable diseases (NCDs), psychological problems, violence, and accident were the main health problems reported among expatriates in previous studies [9–13].

Limited studies have been conducted into health problems among expatriates staying in low-and middle-income countries [4, 6, 13, 14]. The main objective of this review is to describe health problems and gaps in knowledge related to infectious problems and predominant travel-related syndromes among expatriates living in low-and middle-income countries. Results of this review would benefit health practitioners in provision of effective consultation and healthcare for expatriates pre, during, and post travel.

## Search strategies and data analyses methods

MEDLINE database since the year 2000 was searched for relevant literature. Search terms used were “long-term travel”, “expatriate”, and “health problems”. This review includes only English-language articles. The additional references were obtained from hand-searching of selected articles. All relevant information was classified by geographical regions, predominant syndromes, and common diseases including fever, vector borne diseases, sexual transmitted diseases, and animal bite. Exclusion criteria were travel to high income countries, psychological issues, accidents, chronic medical conditions, and dental problem

## Characteristics of long-term expatriates

**Fig. 1:**
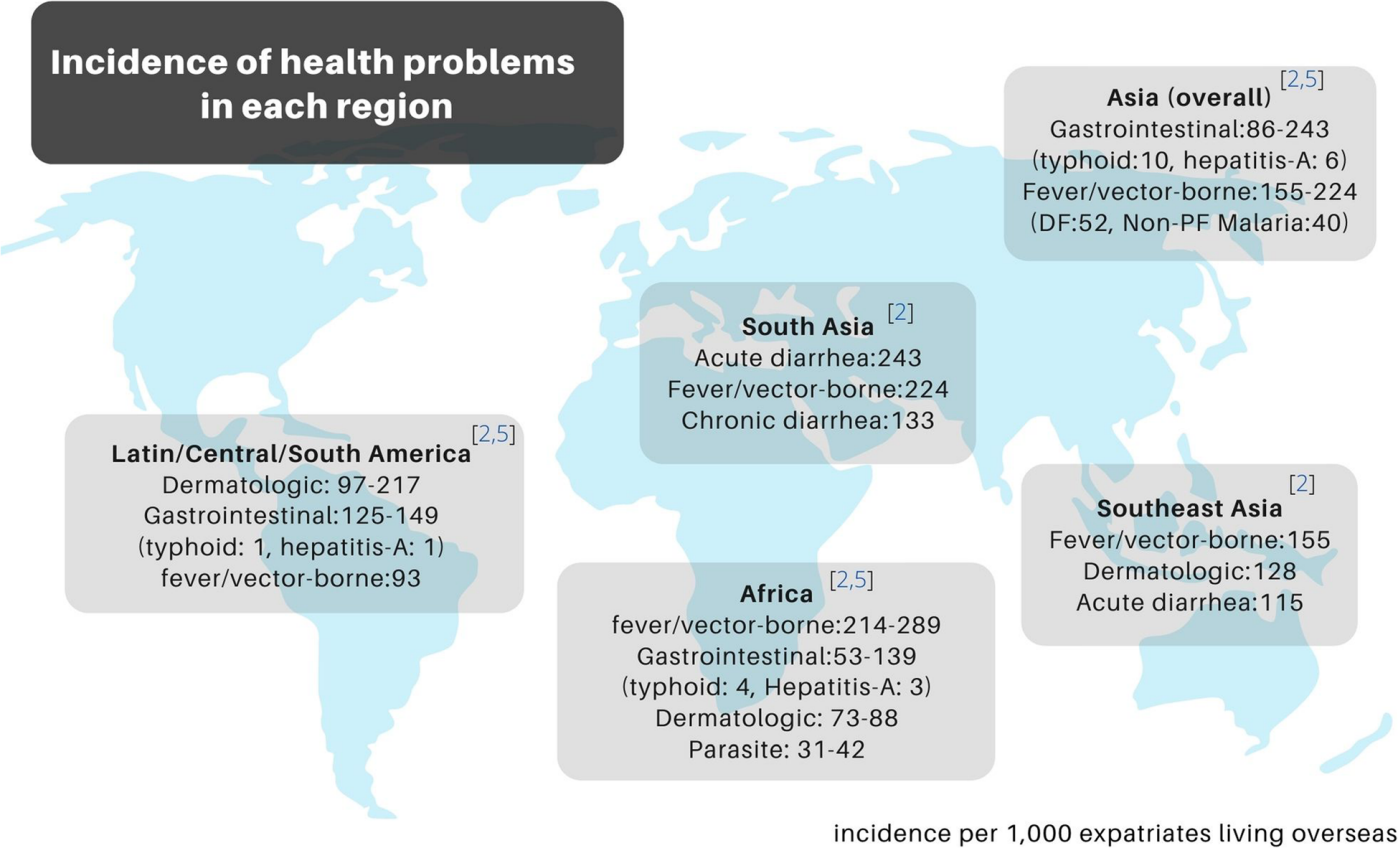
Incidence of health problems reported by destination

The main travel purposes of the expatriates reported in the literatures were volunteer work (40-59%) and business (25-41%) [2, 5]. Most expatriates were from Europe (43%) and North America (29%). Frequent travel destinations were Sub-Saharan Africa (34%), South America (16%), and South-Central Asia (14%) [2]. Most expatriates stayed in destination countries over six months. Between 70 and 73% of expatriates had access to health advice prior to departure. Their sources of health information were as follows, specialists (61.8%), general practitioners (7%), and travel agencies (3%) [2, 10]. The majority (73%) of expatriates experienced at least one medical issue within the first month of staying abroad [10]. Hospital admission and repatriation due to health issues were reported at around 4% and 2% respectively [11]. Only 20% of expatriates remained healthy throughout the whole duration of living overseas. Figure 1 shows a summary of the incidence of common health conditions by destination region.

**Fig. 2:**
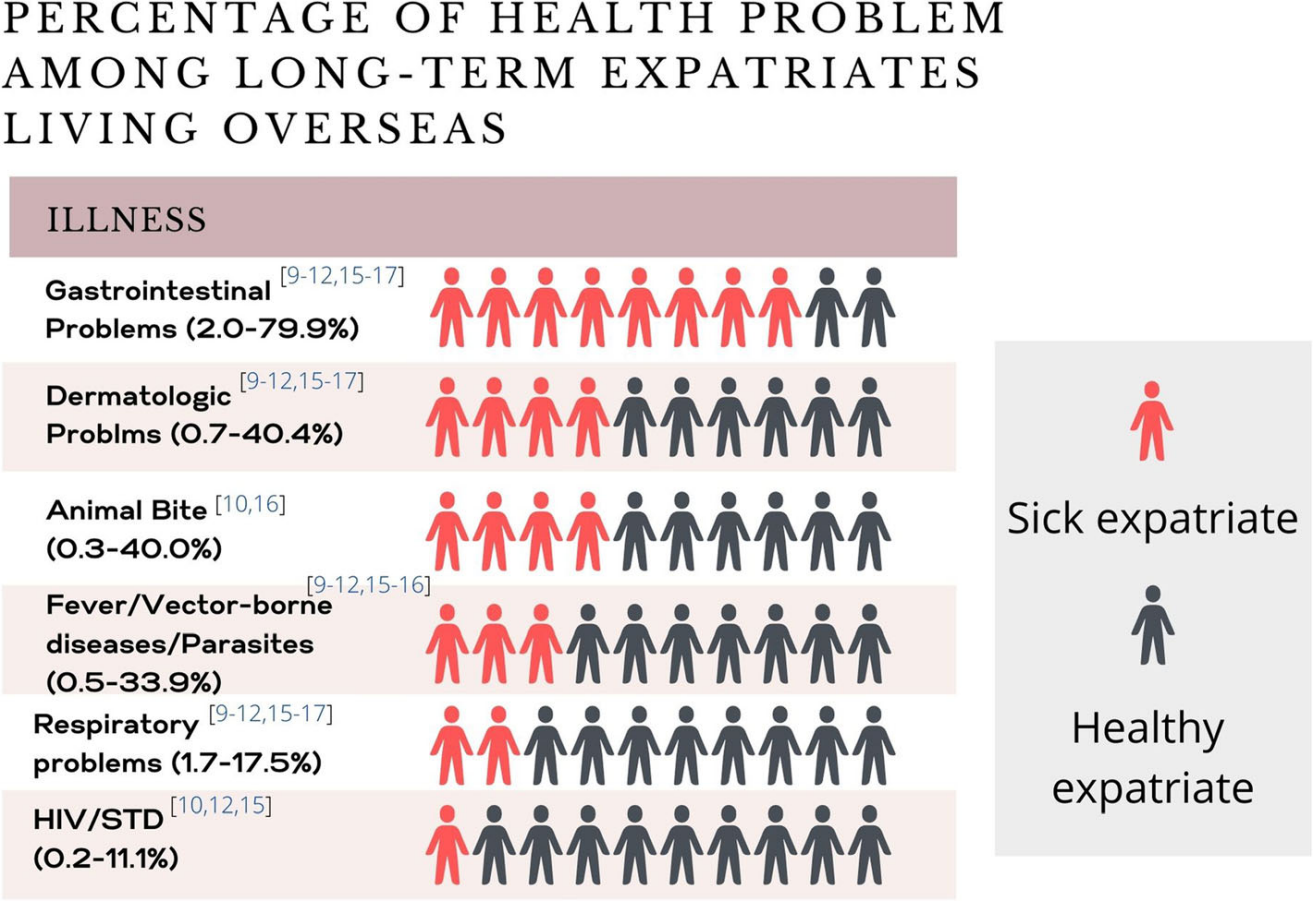
Percentage of Health Problems among Long-term Expatriate Living Overseas

Thirty-seven percent of expatriates reported health problems related to infectious diseases [10]. The majority were minor illnesses and only slightly disrupted work and travel plans. Major health problems reported were vector-borne infections (0.5-33.9%) [10–12, 15–17], sexually transmitted infections (STIs) (0.2-11.1%) [10, 12, 15], and animal bites (0.3-40%) [10, 16] (Fig. 2). The other three predominant syndromes included gastrointestinal problems (up to 80%), dermatologic problems (40%), and respiratory problems (17%) [9–12, 15–17]. .Compared to short-term travelers, expatriates were more likely to be diagnosed with the following diseases: chronic diarrhea (50 per 1,000, OR 1.2, 95% CI 1.04-1.38), *Plasmodium falciparum* (*Pf*) malaria (36 per 1,000, OR 1.5, 95% CI 1.26-1.78), *Plasmodium vivax* (*Pv*) malaria (19 per 1,000, OR 2.5, 95% CI 1.92-3.17), and tuberculosis (11 per 1,000, OR 3.3, 95% CI 2.33-4.56) [2].

### Infectious diseases among expatriates by geographical regions

#### Asia and the Pacific

The incidence of infectious related health problems was 23% among ill returned travelers seen at the GeoSentinel clinic after visiting Asia and the Pacific. Common problems were febrile illnesses (155-224 per 1,000), dengue infection (52 per 1,000), malaria (51 per 1,000), rabies exposure (7 per 1,000), parasitic infections (1-16 per 1,000) and STDs/HIV (5-29 per 1,000) [2, 5, 16]. (Table 1). In the case of syndromic problems, 7.0-50.0% suffered from a gastrointestinal illness (acute/chronic diarrhea, typhoid, hepatitis-A, E infection), followed by respiratory infections (1.7-17.5%), and dermatologic problems (0.9-15.6%). These included bacterial and fungal infections [9, 10, 15, 16, 18, 19]. (Table 2) When compared to other regions of the world, expatriates traveling to Southeast Asia were three times more likely to have latent tuberculosis (TB), with an incidence of 25 cases per 1,000 [2, 5].

**Table 1:** Common Infectious Disease Reported Among Long-Term Expatriates

**Destination**	**Year of study**	**Study population**	**N**	**Health problems**				**Ref**
				**Fever/vector-borne diseases**	**Parasitic**	**STD/HIV**	**Animal bite**	
Asia region								
SEA, Central Asia	2017	Korean	429	-	0.9%	-	-	[9]
SEA	2016	Japanese	209	DF=20.7% Chikungunya=0.9%	-	-	8.6%	[16]
SEA	2009	Long-term travelers	4742	Febrile=155*	5*	7*	-	[2]
South Central Asia	2009	long-term travelers	4742	Febrile=224*	12*	29*	-	[2]
South Asia	2016	Japanese	209	DF=8%	-	-	40%	[16]
East Asia	2016	Japanese	209	-	-	-	15.4%	[16]
Asia Pacific	2012	ill returned expatriates	2883	Febrile=195* DF=52* Malaria non-*Pf* =40* Malaria *Pf* =9* Filaria=6* Rickettsia=5* Leishmania=2*	Tissue parasite=8* Schistosomiasis=1* Strongyloidiasis=16* Ectoparasite=4*	5*	7*	[5]
Asia Pacific	-	European Humanitarian	1190	Febrile=20.6%	-	0.6%	0.3%	[10]
Africa region								
Africa	-	European Humanitarian	1190	Febrile=33.9%	-	0.2%	0.3%	[10]
Africa	2016	Japanese	209	Malaria *Pf* =10%	-	-	3.3%	[16]
Africa	2012	ill returned expatriates	2883	Febrile=289* Malaria *Pf* =115* Malaria non-*Pf* =56* DF=6* Leishmania=3* Rickettsia=4* Filaria=31*	Tissue parasite=14* Amebiasis=31* Giardiasis=33* Schistosomiasis=36* Strongyloidiasis=13* Ectoparasite=5*	5*	2*	[5]
SSA	-	Portuguese	352	Malaria=3.4%	-	-	-	[17]
SSA	2009	long-term travelers	4742	Febrile 214*	42*	28*	-	[2]
Latin, Caribbean, South America								
Latin America	2012	ill returned expatriates	2883	Febrile=95* Malaria *Pf* =1* Malaria non-*Pf* =4* DF=2* Leishmania=15* Filaria=3*	Tissue=14* Amebiasis=42* Giardiasis=14* Schistosomiasis=1* Strongyloidiasis=15* Ectoparasite7*	3*	-	[5]
Central America	2009	long-term travelers	4742	Febrile=108*	-	11*	-	[2]
South America	2009	long-term travelers	4742	Febrile=93*	8*	8*	-	[2]
Overall								
Worldwide		FCO British staff and partners	2020	Febrile=3% Malaria= 1%	2.7%	-	-	[11]
Worldwide	2007	UK returned VSO	219	5.8%	-	11.1%	-	[12]
Worldwide	2007	returning with unsolved health problems	52	-	-	5.8%	-	[12]
Worldwide	2009	long-term travelers	4742	Febrile=154* Malaria *Pf*=36* Leishmania=14*	Giardiasis=36* Schistosomiasis=13* Tissue parasite=6*	-	118*	[2]
Worldwide	2009	missionary expatriates	422	Febrile=20.3% Malaria *Pf* =5% Malaria *Pv*=1.6%	-	3.5%	-	[15]
Worldwide	2009	business expatriates	344	Febrile=21.2% Malaria *Pf* =4.4% Malaria *Pv* =1.9%	-	2.3%	-	[15]
Worldwide	2016	Japanese	209	DF=12.4% Malaria *Pf* =1.4 Chikungunya=0.5%	-	-	12.4%	[16]

Abbreviations: *DF* dengue fever, *STD* sexually transmitted disease, *HIV* Human immunodeficiency virus, *Pf Plasmodium falciparum*, *Pv Plasmodium vivax*, *VSO* Voluntary service overseas, *FCO* Foreign and Commonwealth Office, *GI* gastrointestinal, *SEA* Southeast Asia, *SSA* Sub-Saharan Africa

**Table 2:** Predominant Syndromic Infectious Disease Reported Among Long Term Expatriates

**Destination**	**Year of study**	**Study population**	**N**	**Health problem**			**Ref**
				**Dermatologic**	**Respiratory**	**Gastrointestinal**	
Asian region							
SEA+Central Asia	2017	Korean	429	5.8%	17.5%	7%	[9]
SEA	2016	Japanese	209	0.9%	ARI=9.5% Flu=3.4% Pneumonia=1.7%	36.2%	[16]
SEA	2009	long-term travelers	4742	128*	46*	Acute diarrhea= 115* Chronic diarrhea=86*	[2]
South Central Asia	2009	long-term travelers	4742	94*	38*	Acute diarrhea= 243* Chronic diarrhea= 133*	[2]
South Asia	2016	Japanese	209	-	ARI=8% Pneumonia=4%	32%	[16]
East Asia	2016	Japanese	209	-	ARI=7.7% Flu=3.8% TB 3.8%	50%	[16]
Asia Pacific	2012	ill returned expatriates	2883	94*	Flu= 9* Latent TB=25* Active TB=3* Other 45*	Acute diarrhea= 147* Typhoid=10* Hepatitis-A=6* Hepatitis-E=3*	[5]
Asia Pacific	-	European Humanitarian	1190	15.6%	7.3%	39.4%	[10]
**African region**							
Africa	-	European Humanitarian	1190	17.4%	8.8%	52.5%	[10]
Africa	2016	Japanese	209	-	ARI=13.3% Flu=6.7%	26.7%	[16]
Africa	2012	ill returned expatriates	2883	73*	Flu=6* Latent TB=13* Active TB=3* Other=45*	Acute diarrhea=139* Typhoid=4* Hepatitis-A=3* Hepatitis-E=5* GI, bacteria=9*	[5]
SSA	2009	long-term travelers	4742	88*	41*	Acute diarrhea=107* Chronic diarrhea=53*	[2]
SSA	-	Portuguese	352	-	ARI=4.2% TB=0.3%	GI, other= 2.3% Typhoid=1.4%	[17]
Latin America	2012	ill returned expatriates	2883	103*	Flu=3* Latent TB=15* Active TB=5* Other 23*	Acute diarrhea=129* GI, other=136* Typhoid=1* Hepatitis-A=1* Hepatitis-E=1* GI, bacteria=12*	[5]
Central America	2009	long-term travelers	4742	97*	22*	Acute diarrhea=149* Chronic diarrhea=208*	[2]
South America	2009	long-term travelers	47442	217*	18*	Acute diarrhea= 125* Chronic diarrhea=133*	[2]
**Overall**							
Worldwide	-	FCO British staff and partners	2020	0.7%	1.7%	2%	[11]
Worldwide	2007	UK returned VSO	219	40.4%	-	79.9%	[12]
Worldwide	2007	returning with unsolved health problems	52	15.4%	1.9%	25%	[12]
Worldwide	2009	long-term travelers	4742	118*	Other 38* TB 11*	Acute diarrhea= 133* Chronic diarrhea=23*	[2]
Worldwide	2009	missionary expatriates	422	13.3%	-	20.3%	[15]
Worldwide	2009	business expatriates	344	11.1%	-	20.1%	[15]
Worldwide	2016	Japanese	209	Rubella=2% Measles=0.5%	ARI=11% Flu=3.3% Pneumonia=1.9% TB= 0.5% Other=16.7%	Typhoid 1% Hepatitis-A=1% GI, other=38.8%	[16]

*per 1000, Abbreviations: *ARI* acute respiratory tract infection, *FCO* Foreign and Commonwealth Office, *GI* gastrointestinal, *SSA* Sub-Saharan Africa, *SEA* Southeast Asia, *TB* tuberculosis, *VSO* Voluntary Service Overseas, *UK* United Kingdom

Diarrhea was a common problem particularly among young female (<20 years old) expatriates [2, 12, 13]. Acute diarrhea was reported at 115-243 cases per 1,000 expatriates while chronic diarrhea was lower (86-133 cases per 1,000) [2, 5]. Typhoid fever and gastrointestinal parasites were mainly found in travelers to South-Central Asia. The attack rate was 10 and 8 per 1,000 expatriates, respectively [15] Hepatitis-A was reported at an incidence of only 6 per 1,000 travelers among ill-returned expatriates visiting the Geosentinel clinic [2, 5]. The remainder experienced food poisoning and dysentery [12, 20]. One epidemiologic study of diarrhea in travelers to Thailand reported that Australians and New Zealanders were the most common ethnic group suffering from diarrhea (16%), followed by Europeans (8%) and Americans (7%). The habit of eating outside was linked to an increased risk of diarrhea. The likelihood of this event usually began within the first two weeks of their arrival at their destination [21].

Up to 84% of cases of diarrhea among expatriate workers were associated with parasitic infection [20]. Giardiasis is the most common identifiable parasitic infection found in the Middle East (OR 3.27, *P*-value < 0.05) and South-Central Asia (OR 1.87) when compared to other continents [2]. The prevalence of intestinal parasites among expatriates was 15% [22]. The most common intestinal parasite found was *Giardia* spp. at around 22%, followed by *Entamoeba* spp. (18%), *Trichuris* spp. (16%), *Ascaris* spp (16%), and Hookworm (13%) [22].

Dengue infection is the most common vector-borne infection in Asia, especially prevalent in Southeast Asia [15]. The incidence rate among Dutch expatriates living in Asia was 30 per 1,000 person-months of stay [23], while the Geosentinel network revealed a lower incidence (17 cases per 1,000 long-term travelers) [2]. The seroconversion rate was 6.7% among Israeli travelers who stay at least three months in Asia [24, 25]. Non-*pf* malaria was the second most common vector-borne infection with an incidence of 40 per 1,000 ill returned expatriates [5].

The risk of rabies exposure among expatriates increased with longer duration of stay [2]. Seven percent of Norwegian missionaries who worked in low- and middle- income countries for 4-5 years reported rabies exposure [26]. The incidence of an animal bite, scratch, and lick were 1.7, 1.8, and 6.9 per 1,000 person-months among Japanese expatriates who lived in Thailand, respectively (mainly from dog, cat, and monkey). Fifty-four percent of Japanese expatriates who were bitten by a rabid animal did not seek proper treatment [27]. Moreover, only 10%-34% of expatriates living in Asia received appropriate rabies post-exposure prophylaxis after exposure to rabid animals [28, 29].

### Africa

Approximately 40% of ill return expatriates from African countries reported new health problems [5]. Common travel destinations for expatriates were sub-Sahara African countries such as Angola, Mozambique, Zambia, Zimbabwe, Uganda, Malawi, and Tanzania [2, 5, 10, 16, 17]. The minority traveled to North, West, and Central Africa [5, 18, 30]. Approximately 214-289 per 1,000 expatriates who stayed in Sub-Saharan Africa (SSA) suffered from febrile systemic illness such as *pf* malaria (115 per 1,000), filariasis (31 per 1,000), schistosomiasis (from *S.mansoni* and *S.haematobium*, 36 per 1,000), HIV infection (5-28 per 1,000), rickettsiosis (4 per 1,000), and leishmaniasis (3 per 1,000), (Table 2) [2, 5, 15, 17, 30, 31]. Eosinophilia was found to be in a significantly higher proportion in long-term travelers with an OR = 4.1, 95% CI (2.5-6.8) followed by schistosomiasis (OR = 3.1, 95% CI (2.1-4.6)) and tuberculosis (OR = 2.4, 95% CI (1.1-5.3) when compared to short-term travelers [2]. During a three-year Portuguese mission in Angola and Mozambique, twenty percent of new health complaints or requests of medical attention were infection-related. Of these, 5% needed hospital admission [17]. In the case of syndromic diseases, gastrointestinal problems were reported at a rate of 53-139 per 1,000, followed by dermatologic problems at 73-88 per 1,000, and respiratory problems such as acute respiratory tract infection and TB at 6-41 per 1,000 expatriates [2, 5].

Human African trypanosomiasis with cutaneous lesions (chancre) or central nervous system involvement was reported in expatriates who traveled to East African countries including Uganda, Tanzania, Malawi, Zambia, and Zimbabwe [31]. Only 21 cases of long-term expatriates were identified and evacuated to South Africa for treatment, of these 38% were occupational-related exposure, e.g., military, business, game ranching, or conservation [31]. Non- native African patients were reported in this study which accumulated information for over 14 years [31].

In general, the risk of getting malaria in Africa was three to four times higher compared to other continents [30]. Living in Africa for more than three months increases the risk of malaria infection four-fold [30]. Therefore, Malaria was a common problem among expatriates living in Africa. A study into ill-returned Voluntary Service Overseas (VSO) reported 12% malarial infection and around 38% of them had symptoms during their travel [12, 30]. Most malaria patients acquired the infections in sub-Saharan Africa [30]. *Pf* was the most prevalent species, followed by *P. vivax* and *P.ovale*. In 2001, a lower rate of malarial infection (8 per 100 PCVs-years) was reported among 8,000 US Peace Corps Volunteer (PCV) serving for two years in Madagascar [32]. A recent study from 1996-2008 by the GeoSentinel clinics showed the incident of Malaria among ill-returned travelers from sub-Saharan Africa to be 68 per 1,000 long-term travelers [2]. However, less than 2% of expatriates had good compliance to malaria chemoprophylaxis in their daily living [14] and 62.5% of ill-returned travelers from tropical countries had poor regime in taking chemoprophylaxis medication during their stay [30]. The International Committee of the Red Cross (ICRC) showed better compliance for malaria prophylaxis medication among specialist and delegate groups [10].

Filarial infection was reported in only 0.62% of travelers who visited the GeoSentinel Surveillance network. This was equal to 31 per 1,000 tong-term travelers [2]. Most patients were immigrants who visited friends and relatives (VFR) while the rest were non-urban expatriates. *Onchocerca volvulus* was the most common causative organism which accounted for 37%of the infections, followed by Loa Loa (25%), and *Wuchereria Bancrofti* (25%). The average timing of filarial infection was 125 days after arrival. Within one month of arrival, *O.volvulus* was regularly detected, whereas *L.loa* took 1-6 months. The longest lasting species was *W. bancrofti* (> 6 months*)* [33]. Patients who were infected in Northern Africa and Sub-Saharan Africa accounted for 75% of total infections [33]. Filarial infection was more likely to be discovered among non-endemic visitors due to the popularity of the destinations [33].

Many factors drove the desire of expatriates to have sexual contact during their stay overseas. These included their young age, single status, willingness to have sex prior to the travel, and feelings of boredom and loneliness [34]. Almost one-third of the ICRC expatriates and 41% of Dutch expatriates engaged in casual sex with local partners [10, 34]. More than half of both groups also reported ever paying for sex [34]. Only 64% reported using condoms consistently with casual and steady local sexual partners [10, 12, 34]. The incidence of STIs was 7% among PCVs in Madagascar. Male expatriates had a greater likelihood than female expatriates of contracting STIs (67% vs 33%) [35]. Among Peace Corps male volunteers, consistent condom use was associated with low alcohol consumption and awareness that HIV was a significant health risk [32]. One piece of research found that expatriates who had African sexual partners had a six-fold increased risk of HIV infection [36]. Furthermore, having more than ten sexual partners increased the risk of HIV infection by 14 times [37].

#### Caribbean, Latin America, and South America

Expatriates who visited Caribbean, Latin American, and South American regions displayed similar infectious diseases found in people who traveled to Asian and African countries. Febrile systemic infection was found in 93-108 cases per 1,000 expatriates and dengue infection was reported in 56 cases per 1,000 expatriates living in Lain America [5]

Dermatologic problems and gastrointestinal problems, such as chronic diarrhea and acute diarrhea, were found among returned expatriates at rates of 97-217 per 1,000 cases, 133-208 per 1,000 cases and 125-149 per 1,000 cases, respectively [2, 5]. German expatriates who worked in this region for at least nine years showed a prevalence of hepatitis E infection of around 8.8% [18]. Respiratory problems were the least common (3-23 per 1,000 cases) [2, 5]

The most prevalent vector-borne illness was cutaneous leishmaniasis (15 per 1,000) [2, 5]. When compared to short-term tourists, cutaneous leishmaniasis was shown to be 9 times higher among long-term expatriates visiting the GeoSentinel clinic between 1996 and 2006 [2, 5]. Non-*pf* malaria and dengue infection were less common in this area (4 VS 2 per 1,000 expatriates) [2, 5, 15, 38]. The most frequent parasitic infection reported among ill-returned expatriates from the region was amebiasis (42 per 1,000), followed by strongyloidiasis (15 per 1,000), and giardiasis (14 per 1,000). Reports of *Giardia* spp. was less common when compared to other continents [2]. HIV/STDs appeared in 3-11 cases per 1,000 expatriates [2, 5, 15].

## Discussion

Gastrointestinal health problems were the most prevalent syndromic disease among expatriates. Bacterial and viral pathogens were the most common causes of acute diarrhea, while parasitic infections like giardiasis and amebiasis took a longer time to manifest symptoms. Even though complete adherence is difficult to maintain, basic food and water precautions must be emphasized to expatriates. To reduce the risk of gastrointestinal infections, pre-travel immunizations should be promoted to the travelers as a health precaution. The need for a typhoid vaccine pre-departure should be emphasized to any expatriates who are travelling to Asia, Africa, and Latin America even if its efficacy is only 50-70% [2, 39]. Moreover, hepatitis A vaccine should be recommended to any travelers who plan to visit low to middle income countries.

There is a need for awareness with regards to the danger of importing dengue and malaria in cases among sick travelers returning from Southeast Asia and Africa. The incubation period could help in differential diagnosis, for example a 10-day incubation period could indicate dengue infection [40]. In terms of dengue infection, more seroprevalence studies are needed in other dengue-endemic regions to understand the magnitude of this disease. The duration of stay in the endemic area increased the risk of vector-borne illness. The longer stay, the greater likelihood of visiting remote and rural regions, as well as the increased risk of mosquito bites.

Improved adherence to malaria chemoprophylaxis along with proper use of personal preventive measures are the key to avoiding malaria infection while traveling to endemic areas. In the pre-travel advice, a standby malaria chemoprophylaxis medication with a rapid malarial test kit should be considered among a high-risk population who live in a remote area and have difficulty seeing a doctor. Residing in most of the large cities in SEA was considered as low to no risk of malaria infection because the Anopheles mosquito was less likely to live in the city area, also the mean annual parasite index (API) of all countries in SEA was less than 10 (considered as low malaria transmission risk areas). The API was calculated using the number of new confirmed malaria cases per 1,000 individuals in a specific year [41].

Travel medicine practitioners must be aware of precautions necessary against mosquito and other vectors/insects to grasp all barriers/limitations and give sufficient pre-travel counseling to expatriates. Japanese encephalitis vaccine should be considered in expatriates who plan to reside in the Southeast Asian region, especially if living in rural or suburban areas even if the disease incidence is low (approximately < 1 in million) [6, 9, 42].

In the case of rabies, it was reported that 54% of Japanese expatriates were not obtaining proper rabies post-exposure prophylaxis (PEP) after being bitten by rabid animals [27]. Rabies risk should be included in pre-travel consultations to raise awareness, and clarify misunderstandings, which would lead to adequate care following exposure to any rabid animals. Rabies pre-exposure prophylaxis would be beneficial as a primary prevention especially among expatriates who plan to stay in low- and middle- income countries for a long time.

Falciparum malaria was the most common infectious disease, whereas diarrhea was the most common syndromic condition among long-term expatriates over the last two decades. Overall, other diseases which were prevalent among expatriates such as HIV-infection, dengue and parasitic infection were also on the decline. This circumstance may reflect the fact that most expatriates had better health preparedness and practiced preventive measures while living abroad [2, 3, 5]. Cumulative risk of exposure to illnesses combined with various changes in activities and interaction with local people might result in a wide range of health problems for the expatriates. Predeparture health preparation should be highlighted to reduce potential infectious health hazards [12]. Travel medicine practitioners should be familiar with all requirements, routines, and recommended vaccinations for the travelers. They should know about the availability of the vaccines in their own country as well as additional vaccines accessible in the destination countries. Health practitioners need to update their knowledge frequently from relevant medical research, WHO, US CDC, and other relevant international medical societies as vaccine recommendations are often changed for each destination based on new epidemiological data

To prevent potential infectious disease outbreaks among local people and expatriates, effective surveillance and pre-departure medical screening are essential. Increased social awareness, which can be achieved by training all potential expatriates, is a critical first step toward reducing the risk of acquiring the illnesses. Individual travel risk profile should be assessed so that the travel medicine practitioners can provide proper care and advice. The ability to provide tailored education with up-to-date information, vaccinations, and chemoprophylaxis medication are based on the health risks of the travelers. These include medical history, living exposure, and the destinations. This could potentially prevent the majority of specific and breakthrough infections during their stay until their journey's end [2, 4]. It is not necessary for asymptomatic expatriates returning from their mission abroad to undergo specific laboratory health screening. It is only if the expatriate becomes unwell does screening need to take place. If illness is suspected history taking, targeted physical examination, and a basic set of laboratory tests are recommended. Before thinking of unusual diseases, common infections that are related with symptoms, signs, and specific travel history should be considered first [4].

This review describes frequently reported travel-related syndromic disorders as well as the most common infectious diseases among expatriates residing in low- and middle- income countries, segmented by geographical location. Pre-travel health consultations, education, and vaccinations should be prioritized to reduce the risk of illness and improve disease and symptom awareness among expatriates working or living abroad. This study had some limitations, such as not assessing potential bias and publishing quality. Future review should assess bias and quality of selected articles and focus on the magnitude of diseases or syndromic disorders in specific geographical regions. Standardized categories of exposure and travel-related illness could offer easy ways to compare and follow-up on health problems among expatriates.

## Conclusion

Infectious diseases were major health problems among expatriates traveling to low-and middle-income countries. A detailed knowledge of prevalent infectious diseases and travel-related health problems for particular destinations is essential for proper pre-travel consultation and post-travel diagnosis and care. Travel medicine practitioners must provide individualized education, immunizations, health screenings, and chemoprophylaxis to effectively decrease health risks. Differential diagnosis based on relevant evidence would benefit the ill-returning expatriates to have a better health outcome. More studies on travel-related health issues among expatriates are required since the available information is still limited.

## Data Availability

There is no data set available since this is a review article.

## Abbreviations

API: annual parasite index
CDC: Centers for Disease Control and Prevention
CI: confident interval
HIV: Human Immunodeficiency Virus
ICRC: International Committee of the Red Cross
NCDs: non-communicable diseases
OR: odds ratio
PEP: post-exposure prophylaxis
PCV: Peace Corps Volunteer
Pf: *Plasmodium falciparum*
Pv: *Plasmodium vivax*
STIs: sexually transmitted infections
SSA: Sub-Saharan Africa
TB: tuberculosis
VRF: visited friends and relatives
VSO: Voluntary Service Overseas
WHO: World Health Organization
